# Protein kinase-related tumors in the pediatric population: Updated review on an emerging group with emphasis on the more rarely involved kinases

**DOI:** 10.1007/s00292-026-01537-y

**Published:** 2026-01-28

**Authors:** Uta Flucke, Yvonne M. H. Versleijen-Jonkers, Thomas Mentzel, Annette M. Mueller, Laura S. Hiemcke-Jiwa, Rita Alaggio

**Affiliations:** 1 Princess Maxima Center for Pediatric Oncology, Utrecht, The Netherlands; 2 Department of Pathology, Radboudumc, Geert Grooteplein Zuid 10, 6525 GA Nijmegen, The Netherlands; 3 Department of Medical Oncology, Radboud University Medical Center Nijmegen, Nijmegen, The Netherlands; 4 Kressbronn, Germany; 5 Center of Pediatric Pathology and Pathology, University Hospital Cologne, Cologne, Germany; 6 Department of Pathology, University Medical Center Utrecht, Utrecht, The Netherlands; 7 Pathology Unit, Bambino Gesù Children’s Hospital IRCCS, Rome, Italy; 8 Department of Medico-Surgical Sciences and Biotechnologies, Sapienza University, Rome, Italy

**Keywords:** Soft tissue neoplasms, Immunohistochemistry, Mesenchyme, Mutation, Genetics, Protein kinases, Weichgewebetumoren, Immunhistochemie, Mesenchym, Mutation, Genetik, Proteinkinasen

## Abstract

Advanced and widespread molecular techniques have deepened our understanding of mesenchymal lesions, revealing considerable overlap among morphologically defined entities now known to be related to protein kinases (PKs). This paradigm shift is important for understanding oncogenesis and also in terms of treatment options and prognosis. Therefore, it is preferable to stratify these tumors molecularly instead of morphologically, as the different categories have clinical implications. Molecular analyses are an essential and integrated part of the diagnostic workup of tissue specimens, especially those of young patients. Involved PKs range from receptor tyrosine kinases (neurotrophic tyrosine receptor kinase [NTRK]1, 2, 3; anaplastic lymphoma kinase [ALK]; proto-oncogene 1 [ROS1]; proto-oncogene [RET]; and proto-oncogene/hepatocyte growth factor receptor [MET]; etc.) to intracytoplasmic serine/threonine kinases (RAF proteins) activating the same pathways. Morphological patterns vary from infantile fibrosarcoma(-like) to lipofibromatosis(-like), dermatofibrosarcoma protuberans(-like), and malignant peripheral nerve sheath tumor-like. However, there is considerable overlap histopathologically and immunohistochemically. Most of the neoplasms are (myo)fibroblastic in type, consisting of monomorphic cells. A hemangiopericytoma-like vasculature can be a diagnostic clue. The immunophenotype is characterized by variable expression of smooth muscle actin (SMA)/desmin/CD34 or CD34/S100. This review provides updates to understand the currently known spectrum of PK-related lesions, with emphasis on those occurring more rarely, to aid proper diagnoses and treatment. The aim is to contribute to a better holistic classification.

## Abstract (de)

Neue Methoden zur Detektion molekularer Tumorprofile haben unsere Erkenntnisse zu mesenchymalen Tumoren enorm erweitert und Verwandschaften zwischen einzelnen Entitäten aufgezeigt, die man heute als Proteinkinase(PK)-assoziierte Tumoren zusammenfassen könnte. Dieser Paradigmenwechsel verdeutlicht nicht nur die Onkogenese, sondern beeinflusst auch Therapie und damit die Prognose. Aufgrund der klinischen Relevanz ist es sinnvoll, diese Tumoren vorzugsweise genetisch anstatt morphologisch zu stratifizieren, was die molekularen Analysen zu einem wesentlichen Bestandteil der Diagnostik werden lässt, insbesondere bei jungen Patienten. Die beteiligten PK reichen von Rezeptor-Tyrosinkinasen („neurotrophic tyrosine receptor kinase“ [NTRK]1, 2, 3; „anaplastic lymphoma kinase“ [ALK], ROS, RET, MET usw.) bis hin zu intrazellulären Serin‑/Threoninkinasen (RAF-Proteine), die die gleichen Signalwege aktivieren. Die morphologischen Muster variieren vom infantilen Fibrosarkom über die Lipofibromatose und das Dermatofibrosarcoma protuberans bis hin zum malignen peripheren Nervenscheidentumor, wobei erhebliche histopathologische und immunhistochemische Überschneidungen beobachtet wurden. Die meisten Neoplasien zeigen einen (myo)fibroblastischen Phänotyp und bestehen aus monomorphen Zellen. Hämangioperizytomartige Gefäße können ein diagnostischer Hinweis sein. Der Immunphänotyp ist durch eine variable Expression von „smooth muscle actin“ (SMA)/Desmin/CD34 oder CD34/S100 gekennzeichnet. Die vorliegende Übersichtsarbeit aktualisiert den Kenntnisstand des Spektrums der PK-assoziierten Tumoren mit Schwerpunkt auf seltenere Kinasen und soll helfen, eine korrekte Diagnose und Therapie zu ermöglichen. Langfristig trägt dies zu einer besseren, ganzheitlichen Klassifizierung bei.

During recent decades, advanced and widespread molecular techniques have greatly deepened our understanding of mesenchymal lesions, revealing considerable overlap among morphologically defined entities now known to be related to protein kinases (PKs).

This paradigm shift is not only important for understanding the oncogenesis of these tumors but also has a huge impact on treatment and prognosis. Therefore, attempts are made to (sub)classify tumor entities based on molecular findings, thereby opening up the possibility of therapeutically inhibiting the identified activated PK. As such, molecular analyses are an essential and integrated part of the diagnostic workup of tissue specimens, especially of those from tumors of young patients.

However, morphological suspicion/recognition seems to be the first step toward arriving at a diagnosis, as the spectrum of involved kinases is broad, and the aberrations comprise fusion genes, tandem repeat duplications, point mutations, and amplifications—all activating the kinase domain [1–7]. The most typical morphological growth patterns are presented in Table 1. Often, a hemangiopericytoma (staghorn)-like vasculature is present with perivascular hyalinization, which can be a diagnostic clue [5, 8].

**Table 1:** Common morphological, immunohistochemical, and genetic findings of protein kinase-related mesenchymal tumors (modified from Xu et al. [8] and the 2023 World Health Organization criteria for pediatric tumors)

Tumor type	Morphology, immunohistochemistry, genetics
Infantile fibrosarcoma (IFS)	More or less primitive myoid spindle cells with monomorphic oval to tapered nuclei and inconspicuous cytoplasm; arrangement in sheets and fascicles; hemangiopericytoma-like vasculature; variable expression of SMA, desmin, CD34, S100 *ETV::NTRK3*
IFS-like	See IFS; alternative kinase fusion, e.g., *NTRK1, 2, 3; ALK; LTK; RET; EGFR-KDD; BRAF; MET; FGFR1*
Inflammatory myofibroblastic tumor (IMT)	More or less vague bundles of myofibroblastic cells, background loose–collagenous, SMA ±, desmin +/−, ALK +/− *ALK* (~50–70%), *ROS1, RET, NTRK, PDGFRB, IGF1R*
IMT-like	See IMT; CD34 +/−, S100 +/−
Lipofibromatosis (LPF)/LPF-like neural tumor (LPF-NT)	Irregular bundles of slender myofibroblastic cells mainly located in subcutaneous fat with a collagenous background, univacuolated small fat cells at the interface, SMA +/−, S100 +/−, CD34 +/− *NTRK1, 2, 3; EGFR; ALK; ROS; RET; LTK; FGFR1; PDGFRB*
Malignant peripheral nerve sheath tumor (MPNST)-like	Often cellular, long fascicles of monomorphic spindle cells, S100 +/−, CD34 +/− *NTRK1, 2, 3; LTK; MET; RET; ALK; ROS; (B)RAF*
Adult fibrosarcoma (FS)-like	Cellular, fascicular, herringbone, S100 −, CD34 +/− For genetic changes, see MPNST-like
Myofibroma/myopericytoma	Myoid spindle cells with oval or tapered nuclei, hemangiopericytoma-like (staghorn) vasculature with hyalinization, SMA +, desmin −/+ *PDGFRB*
Myofibroma/myopericytoma/hemangiopericytoma-like	See myopericytoma; CD34 +/−, S100 +/− *NTRK1, 2, 3; RET*
Dermatofibrosarcoma protuberans (DFSP)-like	Infiltrative storiform growth pattern, monomorphic spindle cells, CD34 +, S100 +/− *EGFR *exon 20*, ALK, FGFR1*
Myxoid neoplasms	Variable (mostly less) cellular, vague bundles/storiform arrangement monomorphic spindle and/or epithelioid cells set in a myxoid stroma *NTRK1, 2, 3; EGFR ex 20; ALK; RET; FGFR1*
Epithelioid neoplasms	Sheets/nests epithelioid/ganglion-like cells/histiocytoid, SMA +/−, desmin +/−, CD34 +/−, S100 +/− *NTRK1, 2, 3; ALK; RET*
Fibrous hamartoma of infancy (FHI)	Bundles of spindle cells (between fat), nests/sheets primitive spindle to round cells, fibrous areas with giant cells (giant cell fibroblastoma-like) CD34 +/−, SMA +/− *EGFR* exon 20 mutations
FHI-like	See FHI; S100 +/−, CD34 +/− *MET, FGFR1*

*SMA* smooth muscle actin

*NTRK1-/2-/3-*associated tumors are the prototype and are extensively described in the literature. The first to be identified was the *ETV6::NTRK3* gene fusion in infantile fibrosarcoma and congenital mesoblastic nephroma, which represented a huge step toward the new (sub)classification of such neoplasms (Fig. 1; [5, 9, 10]).

**Fig. 1:**
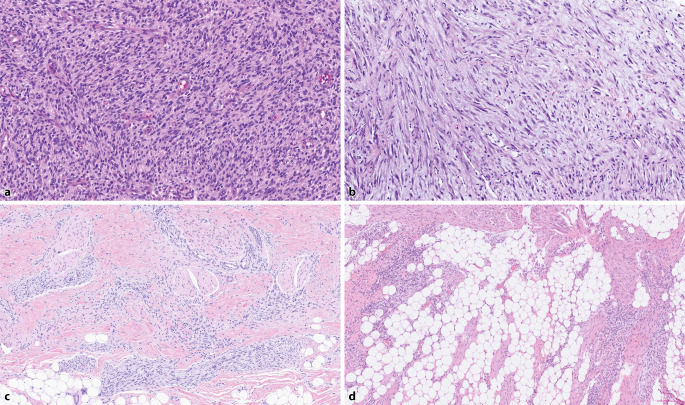
**a** Infantile fibrosarcoma with *ETV6::NTRK3*, ×10 magnification. This classic example shows vague bundles and sheets of primitive myofibroblastic cells with monomorphic nuclei. **b** Inflammatory myofibroblastic tumor with *FN1::ALK*, ×10 magnification. This is a myxoid example consisting of slightly polymorphic myofibroblasts arranged loosely and in bundles, with an obviously abundant amphophilic cytoplasm. **c** Fibrous hamartoma of infancy-like pattern in an otherwise typical infantile fibrosarcoma with *EGFR-*KDD*,* ×5 magnification, comprising nests of primitive cells and bundles of mature slender myofibroblasts. **d** Lipofibromatosis pattern in an otherwise typical infantile fibrosarcoma with *EGFR*-KDD, ×5 magnification

Pathways that become dysregulated by ligand-independent kinase activity with autophosphorylation of tyrosine or serine/threonine are PI3K-AKT, JAK-STAT, RAS-MAPK, CRKL-C2G, and MEKK2/3-MEK5-ERK [11–13].

Involved cells seem to be precursor cells, and, probably due to their developmental stage when the genetic aberration originates, they may be more or less well differentiated, which is morphologically reflected by primitive-looking or more developed neoplasms with a (myo)fibroblastic, pericytic [3, 14], or even vascular phenotype [15]. Primitive cartilage can also be present [4, 16–18].

The aim of this review is to report on the variety of protein kinases involved in the oncogenesis of mesenchymal tumors, mainly of pediatric patients, with a focus on the more rarely documented PKs: EGFR, ALK, LTK, PDGFRB, MET, (B)RAF, RET, FGFR, ABL, and IGF1R. The morphological and immunohistochemical hallmarks of these tumors are presented, including their overlaps, and the usefulness of detecting them is discussed. The presented tabular algorithms aim to provide a helpful overview of this complex and emerging topic (Tables 1 and 2).

**Table 2:** Altered genes encoding for protein kinases and the corresponding morphology

Involved gene	Histology/tumor type
*NTRK1, 2, 3*	IFS(-like), IMT, LPF-like, LPF-NT, adult fibrosarcoma, MPNST-like, myopericytoma-like, epithelioid fibrous histiocytoma/superficial *ALK*-rearranged myxoid spindle cell neoplasm, round cell, epithelioid cell, myxoid neoplasms
*EGFR *exon 20	FHI, LPF, DFSP-like, myxoid spindle cell neoplasm, congenital peribronchial myofibroblastic tumor
*EGFR-*KDD	IFS, LPF-like
*EGFR *rearrangement	LPF/LPF-NT
*EGF*	LPF/LPF-NT
*ALK*	IMT, IFS, LPF-like/LPFNT, DFSP-like, 34+ plaque like dermal fibroma, spindle cell/epithelioid/myxoid neoplasms, epithelioid fibrous histiocytoma/superficial *ALK*-rearranged myxoid spindle cell neoplasm, hemangioma
*ROS*	IMT, LPF-like/LPFNT, MPNST-like
*LTK*	IFS-like, MPNST-like, LPF-like/LPF-NT
*MET*	FHI-like, IFS-like, MPNST-like, LPF-like/LPF-NT
*BRAF/RAF1 (cRAF)*	IFS-like, fibromatosis-like, IMT-like, MPNST-like, round cells
*FGFR1*	IFS-like, LPF-like/LPFNT, DFSP-like, biphasic (primitive/spindle cells), epithelioid (files), myxoid, collagenous
*ABL*	Soft tissue angiofibroma-like, solitary fibrous tumor-like, perineurioma-like
*RET*	IMT, IFS-like, MPNST-like, LPF-like/LPF-NT, myofibroma-like, MPNST-like, epithelioid fibrous histiocytoma/superficial *ALK*-rearranged myxoid spindle cell neoplasm, myxoid, epithelioid/round cell neoplasms
*PDGFRB*	Myofibroma, myopericytoma, dermatomyofibroma, IMT, LPF
*IGF1R*	IMT

*IFS* infantile fibrosarcoma, *LPF* lipofibromatosis, *PPF-NT* lipofibromatosis-like neural tumor, *MPNST* malignant peripheral nerve sheath tumor; *FHI* fibrous hamartoma of infancy, *DFSP* dermatofibrosarcoma protuberans, *IMT* inflammatory myofibroblastic tumor

## *EGFR-*related lesions

The epidermal growth factor receptor (EGFR), a member of the ErbB family of tyrosine kinases, is altered in different mesenchymal tumors. Fusion genes, exon 20 mutations, and kinase domain duplication (KDD) activate the kinase domain and its downstream cascade to induce oncogenesis [3]. Table 3 shows the involved entities with the corresponding genetic aberrations.

**Table 3:** Entities/tumor types and their corresponding *EGFR *alterations

Tumor type	*EGFR* exon 20 mutations	*EGFR*-KDD	*EGFR *rearrangement
Fibrous hamartoma of infancy	X	–	–
Lipofibromatosis	X	X	X
Infantile fibrosarcoma	–	X	–
CD34/S100+ neoplasm	–	–	X
DFSP-like neoplasm	X	–	–
Myxoid spindle cell neoplasm	X	–	–
Mesoblastic nephroma	–	X	–
Congenital peribronchial myofibroblastic tumor	–	X	–

*DFSP* dermatofibrosarcoma protuberans, *EGFR* epidermal growth factor receptor, *KDD *kinase domain duplication

### Fibrous hamartoma of infancy

Fibrous hamartoma of infancy (FHI) was first described in a series by Reye in 1954 and later on confirmed as being a separate entity with benign behavior by Enzinger in 1965, who coined the used term [19].

The lesion mainly arises in male patients with a mean age of 15 months (range birth–14 years) [19–21]. Fibrous hamartoma of infancy is localized subcutaneously, commonly in the shoulder girdle. Other reported sites are the trunk, head and neck region, genital region, pelvic limb girdle, and the (lower) extremities [20, 21]. Changes of the overlying skin (discoloration, hypertrichosis, edema, tethering) are rare [20, 21]. The neoplasms usually grow slowly. Rapid growth is mainly observed in early lesions. Recurrences are identified in a few cases, commonly after incomplete excision but also after a couple of years. Long-term clinical follow-up indicates a benign clinical course [19–21].

Grossly, the lesions are described as being poorly delineated, variably fibrous, and myxoid, including adipose tissue [21].

Histology shows an organoid triphasic pattern characterized by fibrocollagenous trabeculae resembling fetal tendons, desmoid fibromatosis or lipofibromatosis, and nests of primitive mesenchymal cells arranged loosely or in a whorl-like manner in a highly vascularized myxoid matrix. There is haphazardly entrapped fat, with a variable content that can exceed 50% of the tumor mass. In some cases, a variable fibrous stroma that may show slit-like pseudovascular artefacts lined by prominent stromal cells similar to giant cell fibroblastoma is observed [19–21].

A few cases have been reported to show malignant transformation with cellular areas of primitive spindle and round cells with monomorphic nuclei and frequent mitotic figures [21]. Given the EGFR kinase activation in FHI and also in infantile fibrosarcoma (see below), a coincidence of different growth patterns within one tumor is not surprising.

Immunohistochemically, the neoplasms are variably positive for smooth muscle actin (SMA) and CD34, with S100 expression in the fat cells only [20, 21]. While EGFR is reported to be positive in most cases, this marker is not highly consistent and should be used with caution [3, 22].

From a genetic point of view, *EGFR* exon 20 insertion/duplication mutations are identified [3, 22].

Differential diagnoses are lipoblastoma, especially when fibrous or fibromyxoid areas are prominent. However, beside CD34, these cases also express desmin and the pleomorphic adenoma gene (PLAG1) due to *PLAG1* rearrangement [22–24]. Triphasic *MET*-related lesions are another differential diagnosis (see below).

### Other lesions with *EGFR* aberrations

**Fig. 2:**
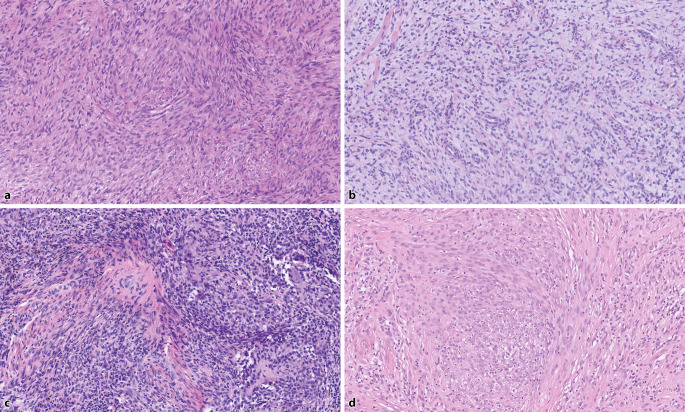
**a** Spindle cell neoplasm with *TPM3::NTRK1*, ×10 magnification, showing vague short bundles and whorls of primitive-looking myofibroblastic spindle cells. **b** Spindle cell neoplasm with *TMF1::RAF1*, ×10 magnification In this myxoid lesion, a loose arrangement of primitive myofibroblastic spindle cells is seen. Note the entrapped collagen. **c** Spindle cell neoplasm with *ETV6::BRAF*, ×10 magnification. The primitive-looking cells are arranged in sheets possessing oval to elongated nuclei. There is a variable fibrous background, an inflammatory reaction, and deposition of hemosiderin. **d** Epithelioid fibrous histiocytoma with *ALK *rearrangement comprising epithelioid and plump spindle cells with round to oval nuclei and an amphophilic cytoplasm. Note the collagenous background

Other lesions with *EGFR *exon 20 mutations are lipofibromatosis (characterized by bundles of myofibroblastic cells with tapered nuclei traversing adipose tissue), dermatofibrosarcoma protuberance (DFSP)-like (with storiformly arranged myofibroblasts), and myxoid spindle cell neoplasm (showing a multinodular growth pattern of monomorphic spindle cells set in a myxoid matrix; Fig. 2; [3, 25]).

*EGFR *kinase domain duplication (KDD) has been found in mesoblastic nephroma, infantile fibrosarcoma, lipofibromatosis-like lesions, and congenital peribronchial myofibroblastic tumor (CPMT). All share morphological features (monomorphic primitive spindle cells arranged in more or less well-defined fascicles). However, the additional presence of primitive cartilage is typical for CPMT and may occur in mesoblastic nephroma and infantile fibrosarcoma [4, 7, 16–18, 26].

*EGFR* fusion genes have been described in lipofibromatosis and also in the case report of a young adult with a CD34-/S100-positive tumor in his lumbar region consisting of confluent nests of slightly pleomorphic polygonal primitive cells. The nests were surrounded by a collagenous stroma [27].

In the molecularly heterogeneous group of lipofibromatosis, *EGF* fusions are also identified, showing that this growth pattern can be associated with the occurrence of different kinases and their ligands [28].

## *ALK*-related lesions

The anaplastic lymphoma kinase (*ALK*) oncogene, located on chromosome 2p23, encodes for a cell surface receptor tyrosine kinase belonging to the insulin receptor kinase superfamily and, together with LTK, to a unique subfamily. It was first discovered as a fusion partner in anaplastic large cell lymphoma [29]. Its recurrent involvement in inflammatory myofibroblastic tumor (IMT) has been known since 1999 [30]. Whereas fusion genes are the predominant genetic aberration, it has been demonstrated that gene amplification, which also results in elevated protein expression, is an alternative mechanism in rare cases [31].

Nowadays it is known that mesenchymal lesions harboring an *ALK* rearrangement show a broad clinicopathologic spectrum, with affection of pediatric and adult patients and occurrence in skin, superficial and deep soft tissue, bone, viscera, and the central nervous system (CNS) [2, 31–33]. However, young patients are more often involved. Beside the well-known entities, such as IMT or epithelioid fibrous histiocytoma, descriptive terms were coined to attribute to the morphology, as all neoplasms have a similar genetic background [34–36]. The tumors reported to date and their features are shown in Table 4. Their morphology varies within and between tumor types, ranging from epithelioid/histiocytoid to myofibroblastic spindle cells organized in sheets, nests, bundles, and whorls in a loose, myxoid, and/or collagenous matrix with possible hyalinization. The nuclei are relatively monomorphic, either ovoid, tapered, or round with prominent nucleoli. The latter is seen in the epithelioid cells possessing also an obvious amphophilic cytoplasm. Lesions with high-grade features have enlarged nuclei, mitotic activity, and necrosis. A hemangiopericytoma-like vasculature can be prominent and therefore diagnostic [32–34, 37]. Immunohistochemically, ALK expression is a perfect surrogate marker. Depending on the partner genes, subcellular localization of the ALK chimeric protein can be membranous, intracytoplasmic, or perinuclear. Other variably expressed markers are SMA, desmin, CD34, and S100 (Table 4; [13, 33]). Exceptionally, cases without *ALK* alteration show ALK expression [38].

Alternatively affected receptor tyrosine kinases are *ROS, RET*, and *NTRK1/2/3*, with *NTRK3* being the prototypical gene involved in oncogenesis of infantile fibrosarcoma. This provides evidence that there is morphological overlap between protein kinase-related lesions [13, 32, 33, 37, 39].

A comprehensive review of *ALK*-rearranged mesenchymal tumors was recently published by Agaimy [33].

### Inflammatory myofibroblastic tumor

Inflammatory myofibroblastic tumor (IMT) is the prototype of an *ALK*-rearranged mesenchymal tumor with myofibroblastic properties and is a member of the intermediate prognostic group (locally aggressive, rarely metastasizing) [33]. It was originally described in the lung under various terms such as inflammatory pseudotumor, plasma cell granuloma, pseudosarcomatous myofibroblastic proliferation, or inflammatory myofibrohistiocytic proliferation. It became apparent that these tumors can also occur at extrapulmonary sites, such as the mesentery and other intraabdominal/retroperitoneal locations, the small pelvis including the genitourinary tract, the head and neck region including the upper respiratory tract and the meninges, the mediastinum including the heart, the breast, and the extremities [40, 41]. The sex distribution of the patients is roughly equal, and the age range is broad, from 3 months to 46 years (mean 9 years) [40, 41]. Patients present with a mass with or without pain. Systemic symptoms are reported in 1/3 of patients, presenting with fever; weight loss; malaise; and/or peripheral blood abnormalities like anemia, thrombocytosis, elevated erythrocyte sedimentation rate, and polyclonal hypergammaglobulinemia caused by cytokine mediators [41].

The tumors can be huge. One large study reports a mean diameter of 6.0 cm (range 1–17 cm). The masses are circumscribed and firm, white or tan, showing a whorled, fleshy, or myxoid cut surface with focal hemorrhage, necrosis, and calcification in a minority of cases [40].

Histology in most IMTs is inhomogeneous and does not correlate with clinical behavior. It can be granulation tissue-like and/or tissue culture-like, resembling nodular fasciitis. Other patterns are compact spindle cell proliferation or scar tissue-like. The constituent myofibroblasts are variable in size and shape and are mainly arranged in vague fascicles. They are spindly or polygonal (ganglion-like), with round, oval, or elongated nuclei and sometimes prominent nucleoli (ganglion-like). The cytoplasm is inconspicuous or abundant and amphophilic (ganglion-like). The stroma may be loose/myxoid and/or collagenous. The inflammatory infiltrate can be more or less prominent, consisting of lymphocytes, plasma cells, eosinophils, and neutrophils [33, 40, 41].

Immunohistochemically, SMA and desmin are variably expressed, and ALK expression corresponds with the *ALK* genetic abnormality. It seems from the literature that up to 70% of IMTs are *ALK-*related [33, 41]. Other involved genes are *ROS, RET, *and* NTRK,* among other rarely involved genes (Table 1; [41]).

Differential diagnosis includes ALK-positive histiocytosis, which shows strong and diffuse CD163-positive staining. PU1.1, a nuclear marker of histiocytes, may be useful to differentiate background histiocytes from fibroblastic/myofibroblastic neoplastic cells in the case of IMT with abundant histiocytes [33].

### Cutaneous hemangioma with epithelioid features and *ALK *rearrangement

These vascular lesions were reported recently. They are exceptional within the group of ALK-related lesions, mostly showing a myofibroblastic or, more rarely, a histiocytic phenotype. The age range of patients is 2–38 years.. Reported sites are the abdominal wall, extremities, shoulder, and head and neck region.

Histologically, lesions are circumscribed, nodular, and surrounded by an epidermal collarette. The vascular channels are lined by epithelioid endothelial cells with round, relatively monomorphous nuclei with fine chromatin and small nucleoli. The mitotic count is 4–6/10 high-power fields (HPFs). Cells have abundant eosinophilic cytoplasm showing focally intracytoplasmic vacuoles. There is maturation toward the periphery, with more distinct vessels lined by flattened endothelial cells. Pericytes are present. There is a chronic inflammatory reaction.

Immunohistochemically, lesions are positive for ERG, CD31, and ALK. Surrounding pericytes are highlighted by SMA. Other markers are negative, e.g., human herpesvirus 8 (HHV8), glucose transporter type 1 (GLUT1), and pankeratin AE. None of the control cases, including epithelioid hemangiomas and epithelioid angiomatous nodules, stained for ALK. All cases harbored an *ALK* fusion gene. Follow-up was uneventful.

Differential diagnoses include epithelioid angiomatous nodule and epithelioid hemangioma harboring *FOS* or *FOSB* rearrangement, with immunohistochemical expression of the corresponding proteins being negative in *ALK*-rearranged hemangiomas. Epithelioid hemangioendothelioma is less likely [13, 15].

**Table 4:** Tumor entities/types with *ALK *rearrangement

Tumor type	Clinical findings	Histology	Positive immunohistochemical results
Inflammatory myofibroblastic tumor*/inflammatory myofibroblastic sarcoma^#^	Children, adults*^#^ Intraabdominal*^#^, intrathoracic*^#^, H&N*, extremities*	(Epithelioid^#^) myofibroblasts, ganglion-like +/− Bundles +/−* Sheets^#^* Collagen +/− Myxoid −/+ inflammation	SMA, desmin, ALK (50–70% of all cases, corresponding to rearrangement)
Infantile fibrosarcoma-like	Children Extremities, H&N, visceral	Haphazardly primitive spindle cells, bundles of myofibroblastic spindle cells, herringbone pattern +/− Collagen +/− Myxoid +/− HPC-like vasculature	SMA +/−, S100 +/−, CD34 +/−
Dermatofibrosarcoma protuberans-like/CD34 + plaque-like dermal fibroma	Children, adults Mainly skin, extremities, limb girdles, trunk, H&N	Infiltrative, storiform, vague bundles, (myo)fibroblastic spindle cells	CD34 + S100 − ALK +
Lipofibromatosis-like	Children, adults Extremities, limb girdle	Sheets, nests, bundles spindle cells infiltrating fat and deeper soft tissue Collagen +/− Myxoid +/− HPC-like vasculature	S100 +/− CD34 +/− ALK +
Spindle cell/epithelioid/myxoid neoplasms	Children, adults Trunk, groin, paratesticular, extremities, H&N, CNS	Sheets epithelioid cells, bundles, whorls, haphazard spindle cells, enlarged or pleomorphic nuclei −/+ Collagen +/− Myxoid +/− HPC-like vasculature	ALK + SMA −/+ Caldesmon −/+ H3K27me3 +/−
Epithelioid fibrous histiocytoma/superficial *ALK*-rearranged myxoid spindle cell neoplasm	Children, adults Extremities, trunk, H&N	Sheets epithelioid cells +/− Round nuclei, binucleation +/− Amphophilic cytoplasm, whorls nests, sheets, bundles histiocytoid spindle cells −/+ Collagen +/− Myxoid +/− (HPC-like vasculature/lipofibromatosis-like)	CD34 +/− S100 −/+ SMA −/+ EMA +/− ALK +
Myxoid fibroblastic tumor of the vocal cord	Adolescents, adults, male>>female	Variable cellularity and variable myxoid background, cytological range: bland-looking spindle cells to epithelioid cells; ganglion-like with enlarged, pleomorphic nuclei, nuclear pseudoinclusions, prominent (capillary) vasculature	SMA +/− S100 − CD34 − Desmin − ALK +
Cutaneous hemangioma with epithelioid features	Children, adults H&N, extremities, trunk	Circumscribed/nodular epithelioid endothelial cells, peripheral maturation	ERG + CD31 + SMA + (pericytes) ALK +

H&N head and neck region, CNS central nervous system, *HPC* Hemangiopericytoma, *SMA* smooth muscle actin, *EMA* epithelial membrane antigen, *ALK* anaplastic lymphoma kinase, *ERG* avian v-ets erythroblastosis virus E26 oncogene homolog

## *ROS1*

The *ROS1 *gene encodes for an RTK that belongs to the sevenless subfamily of tyrosine kinase insulin receptor genes. The resulting type I integral membrane protein is, when altered, mainly involved in the oncogenesis of inflammatory myofibroblastic tumor (IMT; see above). A lipofibromatosis-like/lipofibromatosis-like neural tumor pattern has been reported in exceptional cases [39].

## *LTK-*related lesions

Leucocyte receptor tyrosine kinase (*LTK*) is a member of the *ALK/LTK* family of genes. It has a homology with *ALK* and has been reported to be activated by a fusion transcript upregulating the kinase domain and, consecutively, the PI3K/AKT and MAPK pathways [42].

Three neoplasms were recently described with the identification of a fusion gene. Patients’ age ranged from 3 months to 17 years. Lesions originated in the muscle of the entire leg, the toe, or finger. (Long-term) follow-up was uneventful in two cases. The most extended neoplasm recurred locally.

Grossly, the tumors were described as infiltrative whitish lesions [42]. Histomorphologically, the infiltrative lesions were either composed of sheets of primitive monomorphic spindle cells or more differentiated spindle cells arranged in fascicles within a fibrous background. In two cases, a prominent hemangiopericytoma-like vasculature with perivascular fibrosis was present.

Immunohistochemically, like other PK-related lesions, CD34 and S100 were variably present, with positivity for smooth muscle markers in the more maturated myofibroblastic lesion.

At the RNA expression and methylation levels, cases were similar to DFSP and a tumor with *RAF1* rearrangement, confirming that PK-related lesions are a family of tumors. However, other PK-related tumors like infantile fibrosarcoma and inflammatory myofibroblastic tumors clustered apart. Of note, such a clustering analysis depends on the included entities/cases for comparison and is therefore a matter of perspective [42, 43].

## *MET*-related lesions

The hepatocyte growth factor receptor (HGFR) encoded by *MET *is a tyrosine kinase receptor. It seems to be rarely involved in the oncogenesis of PK-related mesenchymal tumors. Among the four reported tumors, patients’ age ranged from birth to 4 months. Tumors were located superficially and deep: subcutis of the lumbar area, pelvic soft tissue, thigh, trunk, and masseter muscle. The size ranged from 5.2 to 20 cm. Histologically, these tumors have a triphasic pattern like fibrous hamartoma of infancy (see above) and/or an infantile fibrosarcoma‑/MPNST-like appearance. Immunohistochemically, variable expression of S100 and CD34 has been reported. Patients have a good prognosis [39, 44, 45].

## *BRAF/RAF1(cRAF)*-related lesions

The RAF proteins are cytosolic serine/threonine kinases activating the MAPK cascade downstream of RAS. The corresponding genes can be altered by mutations or fusion genes.

### RAF1 (cRAF)

*RAF1*-rearranged tumors have mostly been reported in infants. Tumors were located in the arm, thigh, and kidney, with a size of up to 12 cm. The described cases were primitive looking, with variable cellularity like IFS/CMN (cellular mesoblastic nephroma), or had a more mature myofibroblastic morphology, with a fibromatosis-like appearance showing long fascicles of slender spindle cells. Nuclei were oval or tapered, monomorphous, and bland looking; S100 and CD34 were variably positive [12].

### BRAF

Patients with *BRAF-*altered tumors ranged in age from birth to 32 years. Tumors originated in the soft tissues of the extremities, the small pelvis, and the retroperitoneum including the rectum wall and the kidney as well as in the mediastinum and the spinal, paraspinal, and head and neck regions [4, 17, 46]. When involved, skin can be ulcerated [46]. A neoplasm occurring in bone (mastoid) of a 14-year-old boy has also been described [6].

Lesions showed IFS-like and also IMT-like morphology consisting of monomorphic spindle cells, either primitive or with a more myofibroblastic phenotype possessing oval or elongated/tapered nuclei. A more or less fascicular architecture with infiltrative growth is common. Areas of round cells may be present. In the background, collagen with variable myxoid changes can be variably seen, but it is often scarce combined with high cellularity. The vascular pattern is often hemangiopericytoma-like, which can be a diagnostic clue in receptor protein kinase-related tumors mainly with an IFS-like pattern. Heterologous cartilage may occur [4, 17].

Immunohistochemically, SMA expression is seen to a variable degree, with desmin being positive in exceptional cases; CD34 and S100 positivity is observed in some cases [4, 17].

Most cases harbor a *BRAF* fusion gene. Alternatively, a point mutation can occur, including p.V600D, p.V600E, and p.L485F. Tandem duplications and compound deletions have also been reported. In exceptional cases, a combined *BRAF *fusion or a *BRAF* fusion gene in addition to a point mutation has been identified [4, 7, 17, 46]. Tumors may bear additional mutations, e.g., in *P53* or *APC* [46].

In terms of follow-up, lesions may recur, also after years, but there is no apparent progression after long-term follow-up in most cases. However, aggressive behavior has been documented in isolated cases [17, 46].

Whether there is a link to metanephric stromal tumors [46] needs to be explored more thoroughly.

## *FGFR1*-related lesions

The fibroblast growth factor receptor (FGFR) family comprises the RTKs. Normally, they become activated by binding of fibroblast growth factors. In soft tissue tumors, fusion genes are found, leading to hyperfunctioning of the kinase [1–4].

The reported patients were infants. Tumors were located in deep gluteal or pelvic/perirectal soft tissue, with involvement of the rectal wall. The size of the tumors ranged from 4.2 to 11 cm. Neoplasms were mainly highly cellular. They consisted of primitive spindles arranged in vague bundles, whorls, and/or sheets. Infiltration of the fat was lace-like/DFSP-like. Epithelioid cells arranged in sheets or cords were also present. Less cellular areas were collagenous or myxoid. Cellular atypia was observed after chemotherapy. Mitotic figures were scarce. Immunohistochemical analysis revealed CD34 expression without S100 staining. Patchy desmin expression was observed in rare instances. Locally aggressive behavior has been documented [47].

## *ABL*-related lesions

The ABL family of kinase genes comprises proto-oncogenes 1 and 2 encoding for non-receptor tyrosine kinases localized in the nucleus and cytoplasm. They fuse with a variety of genes in hematological malignancies. Fusion genes in mesenchymal tumors are rare.

Lesions occurred mainly in pediatric patients (age range 7–76 years) and were localized in the trunk, limb girdles, and proximal and distal extremities, superficially or deep. Reported sizes were up to 11 cm. These tumors were misinterpreted as soft tissue angiofibroma, solitary fibrous tumors, and perineurioma.

Histologically, neoplasms are circumscribed or infiltrative and consist of bland-looking monomorphic spindle cells arranged in sheets, bundles, and whorls, with a fibrous/fibromyxoid background. When the cells have long cytoplasmic processes, they resemble perineurial cells. Mitotic activity is low.

Immunohistochemistry reveals positivity for CD34, S100, EMA, GLUT1, and claudin, with the latter three markers being characteristic of perineurioma. However, lesions mainly show morphological, immunohistochemical, and molecular features of PK-related lesions, as mentioned in Table 1.

Locally aggressive behavior seems to be an exception [48, 49].

## *RET-*related lesions

The rearranged during transfection (*RET*) gene encodes a receptor tyrosine kinase for members of the glial cell line-derived neurotropic factor family of extracellular signaling molecules. The *RET* fusion genes are responsible for autonomic activation of the kinase, leading to oncogenesis of mesenchymal neoplasms with the typical characteristics reported for PK-related lesions.

Tumors occur in children, including newborns and infants. Adults may also be affected. Neoplasms range in size from 2.9 to 13 cm. They are located in superficial and deep soft tissue and may affect bone (chest wall). Reported sites are the proximal and distal extremities, gluteal region, trunk, neck, and visceral organs (lung, kidney).

Morphologically, neoplasms are described as IFS-like, MPNST-like, and LPF-like/LPF-NT, with sheets/bundles and whorls of monomorphic primitive spindle cells. The background is variable and can be collagenous or myxoid. A hemangiopericytoma vasculature can be prominent. In immunohistochemistry, expression of CD34, S100, and SMA was variably identifiable. Of note, ALK and ERG expression is possible.

Rarely occurring high-grade morphology with a high nuclear–cytoplasmic (NC) ratio and necrosis correlate with malignant behavior. Metastases can develop in the lung, lymph nodes, soft tissue, or CNS [38, 50].

## *PDGFRB-*related lesions

Platelet-derived growth factor receptor beta (PDGFRB) and its paralog alpha belong to the class III family, along with c‑KIT, colony-stimulating factor 1 receptor, and FMS-like tyrosine kinase 3 receptor. They play a role in various disorders, including soft tissue tumors [51].

### Pericytic tumors

Pericytic tumors comprise myofibroma, myopericytoma, angioleiomyoma, and glomus tumors. Myofibroma, myopericytoma, and angioleiomyoma are on a morphological continuum. The age range is wide, with myofibromas being very common in (early) childhood. Myofibromas occur solitarily or, less commonly, multiply (myofibromatosis) in skin/subcutis, deep soft tissue, bone, and viscera. The latter can be life threatening. Rarely, hereditary tumors are reported [52].

Morphology is typically biphasic in myofibroma, with primitive-looking areas and maturated myofibroblasts in a bluish matrix. Myopericytomas show sheets and concentric growth of primitive or maturated myofibroblasts around blood vessels and a variable collagenous background. In both tumors, an HPC-like vasculature may be obvious. The latter is also characteristic of angioleiomyomas and glomus tumors, surrounded by either leiomyocytes or glomus cells. Such lesions rarely originate in the pediatric population. Immunohistochemically, SMA and desmin are variably expressed [52, 53].

*PDGFRB* point mutations have been reported in all mentioned lesions. They occur in the juxtamembrane (exon 12) or kinase domain (exon 14), leading to activation of the kinase [53].

Myofibromatosis has also been shown to bear a *PDGFRB* fusion gene [54]. Other reported fusion genes are *MTCH2-FNBP4, FN1-TIMP1, COL4A1-VEGFD *(with and without *PDGFRB* mutation) [55].

### Dermatomyofibroma

Dermatomyofibroma, first reported by Hügel in 1992, is a benign skin lesion occurring rarely in children, with neoplasms originating in the neck, shoulder girdle, trunk, and extremities. They are plaque-like and consist of fascicles of uniform slender myofibroblasts with elongated nuclei. The fascicles are parallel to the epidermis. Elastic fibers are preserved, and skin adnexal structures are spared. When infiltrating the subcutis, there are similarities with lipofibromatosis [51, 56].

Immunohistochemically, SMA, calponin, and CD34 may be positive [51, 56]. *PDGFRB* point mutations similar to those seen in pericytic tumors have been identified [51].

### IMT, lipofibromatosis

*PDGFRB *fusions are more rarely documented in IMTs (see above) and also in lipofibromatosis [28, 57].

## *IGF1R-*related lesions

Insulin-like growth factor 1 receptor (*IGF1R*) is a receptor tyrosine kinase that binds insulin-like growth factor. The receptor is involved in physiological and pathological processes. It has an anti-apoptotic effect.

It can be an alternative fusion gene to ALK and has been reported in an inflammatory myofibroblastic tumor occurring in the duodenum of a middle-aged woman [58].

## Conclusion

This review provides an update of the variety of protein kinases involved in the oncogenesis of mesenchymal tumors, mainly in pediatric patients, with a focus on the more rarely documented PKs—EGFR, ALK, LTK, PDGFRB, MET, (B)RAF, RET, FGFR, ABL, and IGF1R. The morphological and immunohistochemical hallmarks of such tumors, including their overlap, are highlighted, in order to facilitate detection of such tumors because of the therapeutic consequences.

## Declarations

For this article no studies with human participants or animals were performed by any of the authors. All studies mentioned were in accordance with the ethical standards indicated in each case.
